# PreCanCell: An ensemble learning algorithm for predicting cancer and non-cancer cells from single-cell transcriptomes

**DOI:** 10.1016/j.csbj.2023.07.009

**Published:** 2023-07-11

**Authors:** Tao Yang, Qiyu Yan, Rongzhuo Long, Zhixian Liu, Xiaosheng Wang

**Affiliations:** a Biomedical Informatics Research Lab, School of Basic Medicine and Clinical Pharmacy, China Pharmaceutical University, Nanjing 211198, China; b Cancer Genomics Research Center, School of Basic Medicine and Clinical Pharmacy, China Pharmaceutical University, Nanjing 211198, China; c Big Data Research Institute, China Pharmaceutical University, Nanjing 211198, China; d Jiangsu Cancer Hospital, Jiangsu Institute of Cancer Research, The Affiliated Cancer Hospital of Nanjing Medical University, Nanjing, Jiangsu Province, China

**Keywords:** Ensemble learning algorithm, Single-cell transcriptomes, Cancer and non-cancer cells, Tumor marker genes, Non-tumor marker genes

## Abstract

We propose PreCanCell, a novel algorithm for predicting malignant and non-malignant cells from single-cell transcriptomes. PreCanCell first identifies the differentially expressed genes (DEGs) between malignant and non-malignant cells commonly in five common cancer types-associated single-cell transcriptome datasets. The five common cancer types include renal cell carcinoma (RCC), head and neck squamous cell carcinoma (HNSCC), melanoma, lung adenocarcinoma (LUAD), and breast cancer (BC). With each of the five datasets as the training set and the DEGs as the features, a single cell is classified as malignant or non-malignant by *k*-NN (*k* = 5). Finally, the single cell is determined as malignant or non-malignant by the majority vote of the five *k*-NN classification results. We tested the predictive performance of PreCanCell in 19 single-cell datasets, and reported classification accuracy, sensitivity, specificity, balanced accuracy (the average of sensitivity and specificity) and the area under the receiver operating characteristic curve (AUROC). In all these datasets, PreCanCell achieved above 0.8 accuracy, sensitivity, specificity, balanced accuracy and AUROC. Finally, we compared the predictive performance of PreCanCell with that of seven other algorithms, including CHETAH, SciBet, SCINA, scmap-cell, scmap-cluster, SingleR, and ikarus. Compared to these algorithms, PreCanCell displays the advantages of higher accuracy and simpler implementation. We have developed an R package for the PreCanCell algorithm, which is available at https://github.com/WangX-Lab/PreCanCell.

## Highlights

• PreCanCell is an algorithm for predicting cancer and non-cancer cells from single-cell transcriptomes.
• PreCanCell is a simple and effective ensemble learning algorithm.
• PreCanCell uses well-annotated and high-quality cancer single-cell transcriptomes as the training set.
• PreCanCell does not require users to provide gene markers and training or reference datasets.
• PreCanCell displays better performance in predicting cancer and non-cancer cells than most established algorithms.

## Graphical Abstract

**Figure:**
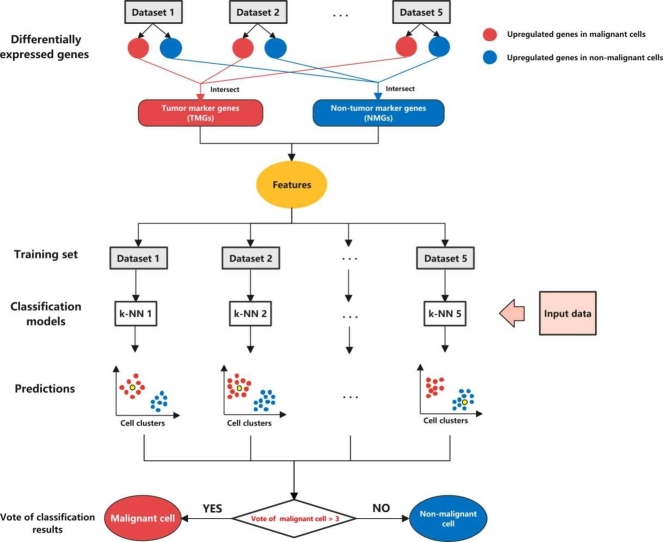

## 1 Introduction

Single-cell RNA sequencing (scRNA-seq) has been widely utilized to characterize transcriptomic profile of single cells [1]. With the emergence of a huge amount of single-cell transcriptomic data generated by scRNA-seq, accurate identification of cell types has become one of the most important and challenging issues in scRNA-seq data analysis [2]. There are two classes of approaches for cell type identification: unsupervised and supervised methods. The unsupervised method identifies cell types by clustering cells based on their gene expression profiles without labeled data [3], while the supervised method infers the identity of each cell by learning of training data with cell type annotation [4]. The unsupervised method is effective in uncovering novel cell populations and in mapping cells from whole organs or organisms [5], [6]. Nevertheless, the cell type annotation with the unsupervised method is often labor-intensive with increased number of cells and samples as it involves manual examination of cluster-specific marker genes. In contrast, the supervised method can fast and automatically annotate cell types by predicting the identity of individual cells. Some commonly used algorithms for identifying cell types included CHETAH [7], SciBet [8], SCINA [9], scmap-cell [10], scmap-cluster [10], SingleR [11] and ikarus [12], where CHETAH, SciBet, scmap-cell, scmap-cluster, SingleR and ikarus are supervised methods, while SCINA is a semi-supervised method. CHETAH annotates cell types by hierarchical classification based on the expression profile of feature genes [7]. SciBet employs the multinomial-distribution model and maximum likelihood estimation to identify cell types [8]. The scmap-cell algorithm assigns cell types by the *k*-nearest-neighbor (*k*-NN) classification based on cosine similarity measure [10], and scmap-cluster classifies a cell based on the greatest similarity between the cell and each cluster centroid or cell [10]. SingleR infers cellular identity based on reference transcriptomic datasets of pure cell types by Spearman correlation [11]. The ikarus algorithm employs a logistic regression classifier along with adaptive network propagation to identify cell types [12]. SCINA assigns cell types by the expectation-maximization model based on the expression profile of user-defined markers [9].

Single-cell transcriptome analysis has been widely employed in investigating various human diseases, particularly in cancer [13], [14], [15], [16], [17]. A prominent task in downstream analysis of cancer single-cell transcriptomes is how to accurately distinguish malignant (or cancerous) from non-malignant cells. A basic principle to resolve this issue is based on the abnormal copy number profile, namely aneuploid, displayed in malignant cells [18]. Many algorithms have recently been developed to infer copy number alteration (CNA) profiles from transcriptome data to distinguish malignant from non-malignant cells [19], [20], [21]. Usually, to perform better inference, these algorithms require sufficient reference information, e.g., aligned BAM files from normal samples. Another class of algorithms to infer malignant and non-malignant cells from transcriptome data are using gene markers for specific cancer types to determine whether a cell is malignant or benign [15], [22].

With a dramatic increase of well-annotated cancer single-cell transcriptomes to date, using the supervised method to infer malignant and non-malignant cells by learning these data may greatly improve the predictive performance. Based on this idea, this study proposed a novel algorithm, termed PreCanCell, for predicting malignant and non-malignant cells from single-cell transcriptomes. We first identified the differentially expressed genes (DEGs) between malignant and non-malignant cells commonly in five cancer single-cell transcriptome datasets. These datasets were associated with several common cancer types, including renal cell carcinoma (RCC), head and neck squamous cell carcinoma (HNSCC), melanoma, lung adenocarcinoma (LUAD), and breast cancer (BC). The number of malignant cells in each of the five datasets is relatively large, with the proportion > 10 %. Using these DEGs as features and the five cancer single-cell transcriptome datasets as the training set, PreCanCell predicts a single cell as malignant or non-malignant based on its gene expression profiles with the *k*-NN (*k* = 5) classifier. We compared PreCanCell with seven other algorithms in several predictive performance, including accuracy, sensitivity, specificality, balanced accuracy (the average of sensitivity and specificity) and the area under the receiver operating characteristic curve (AUROC). We also analyzed correlations of these DEGs with various cancer-related molecular and clinical features in pan-cancer. This study provides a simple but effective method for identifying malignant and non-malignant cells, and a set of reliable cancer and non-cancer marker genes inferred from single cell transcriptomes.

## 2 Methods

### 2.1 Data acquisition and preprocessing

We collected 16 publicly available scRNA-seq datasets, which were gene expression profiles of 663,760 cells. These datasets covered 11 cancer types, including RCC (T1_RCC), HNSCC (T2_HNSCC), melanoma (T3_Melanoma and P11_Melanoma), LUAD (T4_LUAD), BC (T5_BC and P1_BC), basal cell carcinoma (BCC) (P2_BCC), hepatocellular carcinoma (HCC) (P3_HCC and P6_HCC), synovial sarcoma (SyS) (P4_SyS and P5_SyS), glioblastoma (GBM) (P7_GBM and P10_GBM), pancreatic ductal adenocarcinoma (PDAC) (P8_PDAC), and metastatic castration-resistant prostate cancer (mCRPC) (P9_mCRPC). Among the 16 datasets, T1_RCC, T2_HNSCC, T3_Melanoma, T4_LUAD, and T5_BC were the training set, and the others were test sets. In addition, we obtained a scRNA-seq dataset for pan-cancer cell lines (GSE157220) and a scRNA-Seq dataset for immune cells across health human tissues (GSE126030) from the NCBI gene expression omnibus (https://www.ncbi.nlm.nih.gov/geo/). Furthermore, we generated a synthetic scRNA-seq dataset by merging GSE157220 and GSE126030. For cell type annotation, we adopted original methods provided by these datasets-associated publications as ground truth. For all datasets, we performed quality control of reads and cells following the quality control steps described in the original publications. After data quality control, we performed normalization of gene expression values using the following methods. For full-length transcriptome data, gene expression levels were quantified as: *E*_*i,j*_ *=* log_2_(*TPM*_*i,j*_/10 + 1), where *TPM*_*i,j*_ refers to transcript-per-million of gene *i* in cell *j*. For unique molecular identifier (UMI) data, we normalized gene expression values using the “NormalizeData()” function in the R package “Seurat” (v4.0.6) [23] with the default parameters. Namely, the UMI count of each cell was normalized by size-factor 10,000 and then log(*x* + 1) transformation.

To analyze correlations of the DEGs with cancer-related molecular and clinical features in pan-cancer, we downloaded RNA-seq gene expression profiling (RSEM normalized) and clinical data for 33 TCGA cancer types from the genomic data commons data portal (https://portal.gdc.cancer.gov/). We downloaded microarray (Affymetrix Human Genome U219 array) gene expression profiling data for 962 human cancer cell lines and their drug sensitivities (IC50 values) to 265 compounds from the Genomics of Drug Sensitivity in Cancer (GDSC) project (https://www.cancerrxgene.org/downloads). In addition, we downloaded data of RNA-seq gene expression profiling (RSEM normalized) in 1033 cancer cell lines and 51 normal cell lines from the Cancer Cell Line Encyclopedia (CCLE) project (https://depmap.org/portal/download/). All RNA-seq gene expression values (*x*) were transformed by log_2_(*x* + 1) before subsequent analyses. A summary of the datasets used in this study is shown in Supplementary Table S1.

### 2.2 Identification of DEGs between malignant and non-malignant cells

For each training dataset, we identified DEGs between malignant and non-malignant cells. We obtained the DEGs based on the following criteria. First, the gene expression difference between malignant and non-malignant cells was statistically significant if the adjusted Wilcoxon rank-sum test *P*-value < 0.05 by the Bonferroni correction. Second, for a differentially expressed gene, if it was expressed in at least 10 % of malignant cells and had a mean expression fold change of > 0.25 (malignant versus non-malignant cells, log scale), it was defined as an upregulated gene in malignant cells; if it was expressed in at least 10 % of non-malignant cells and had a mean expression fold change of > 0.25 (non-malignant versus malignant cells, log scale), it was defined as an upregulated gene in non-malignant cells. Finally, to credibly identify marker genes for malignant and non-malignant cells, we obtained the gene set, which was the intersection of the upregulated genes in malignant cells identified in each of the five training datasets; this gene set was defined as tumor marker genes (TMGs). The intersection of the upregulated genes in non-malignant cells identified in each of the five training datasets was defined as non-tumor marker genes (NMGs). The DEGs were the set of genes in TMGs or NMGs. We performed the differential gene expression analysis with the function “FindMarkers()” in the R package “Seurat” (v4.0.6).

### 2.3 Classifier development and evaluation

The prediction model PreCanCell utilizes the DEGs as features to predict malignant and non-malignant cells. Before the development and evaluation of classifier, all gene expression values are scaled to the range [0,1] by min-max normalization in each dataset. That is, for each original gene expression value *x*, we scale it as follows:

*x*_*scaled*_ = (*x* − *min*(*x*))/(*max*(*x*) − *min*(*x*)),

where *min*(*x*) and *max*(*x*) denote the minimum value and maximum value of gene expression across all single cells, respectively. With the DEGs as features and *k*-NN (*k* = 5) as the classifier, PreCanCell first predicted malignant and non-malignant cells in each training dataset and reported 10-fold cross-validation results. Next, PreCanCell predicted malignant and non-malignant cells in each test set. In the test set prediction, PreCanCell employs the ensemble learning algorithm. That is, PreCanCell predicts a single cell as malignant or non-malignant with the DEGs as features and *k*-NN (*k* = 5) as the classifier based on each of the five training datasets, and finally assigns the cell by the majority vote of five classification results. The classification accuracy, sensitivity, specificity, balanced accuracy and AUROC were reported.

### 2.4 Evaluation of immune score, stromal score, tumor purity, proliferation score, and intratumor heterogeneity (ITH) score of tumor bulks

We utilized the ESTIMATE algorithm [24] to evaluate the immune score, stromal score, and tumor purity for each TCGA tumor sample based on its gene expression profiles. The immune score, stromal score, and tumor purity represent the immune infiltration level, stromal content, and proportion of tumor cells in the tumor bulk. In addition, we used the single-sample gene-set enrichment analysis (ssGSEA) [25] to assess the proliferation score of a tumor based on the expression profiles of the proliferation marker genes [26] in the tumor. We utilized the DITHER algorithm [27] to measure ITH. DITHER scores ITH based on both profiles of somatic mutations and copy number alterations in the tumor.

### 2.5 Survival analysis

For each cancer type from the TCGA project, we used the median expression levels of TMGs and NMGs as the cut-off value to subgroup samples into low-expression (< median) and high-expression (> median) classes. We employed the Kaplan–Meier (KM) model [28] to compare survival prognosis between two classes of samples. KM curves were used to display survival time differences, whose significance was evaluated by the log-rank test. We implemented survival analyses with the function “survfit()” in the R package “survival.”.

### 2.6 Statistical analysis

In comparisons of the expression levels of TMGs and NMGs between two classes of samples, we used two-tailed Student’s *t* tests. We used the false discovery rate (FDR), which was evaluated by the Benjamini-Hochberg method [29], to adjust for *P*-values in multiple tests. In evaluating correlations between the expression levels of TMGs and NMGs and other variables, we utilized Spearman correlations and reported correlation coefficients (*ρ*) and *P*-values.

### 2.7 Comparisons among different algorithms

We compared the predictive performance (AUROC, accuracy and balanced accuracy) between PreCanCell and seven other algorithms, including CHETAH [7], SciBet [8], SCINA [9], scmap-cell [10], scmap-cluster [10], SingleR [11] and ikarus [12]. Among them, CHETAH, SciBet, SCINA, scmap-cell, scmap-cluster and SingleR were implemented with the R package, and ikarus was implemented with the python package. We ran these tools with the input of normalized scRNA-seq data and with default parameters, except for specific parameters: “allow_unknown = 0” in SCINA and “thresh = 0” in CHETAH. More methodological details for these algorithms are described in Supplementary Table S3. Two algorithms, namely SingleR and PreCanCell, were implemented with multiple workers for parallel execution to evaluate their computational resources. The maximum CPU usage (%), maximum memory usage (%) and running time (seconds) were reported for each algorithm in each of the 11 test sets. The CPU usage (%) represents the share of CPU time used by the process since the last update, and the memory usage (%) the share of physical memory used by the process. The running time indicates the time from the start to the end of running a task.

## 3 Results

**Fig. 1:**
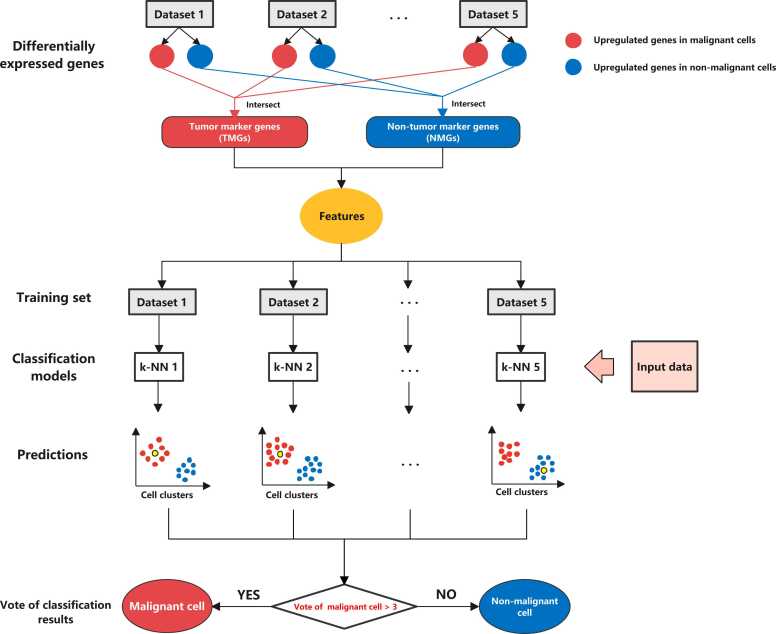
A schematic illustration of the algorithm PreCanCell.

PreCanCell is a method for distinguishing between malignant and non-malignant cells based on their gene expression profiles. This method first identifies the DEGs between malignant and non-malignant cells in common in five single-cell transcriptome datasets (Table 1). With each of the five single-cell transcriptome datasets as the training set and the DEGs as the features, a single cell is classified as malignant or non-malignant based on its gene expression profile by the *k*-NN (*k* = 5) classifier. Finally, the single cell is classified by the majority vote of the five *k*-NN classification results. Fig. 1 is a schematic illustration of PreCanCell. This algorithm has been developed into a R package for public use, which is available at Github (https://github.com/WangX-Lab/PreCanCell). Here we first analyzed correlations between these DEGs’ expression and cancer-related molecular and clinical features in pan-cancer and in individual cancer types. Next, we reported the predictive performance of PreCanCell in 16 datasets. Finally, we compared the predictive performance of PreCanCell with that of seven other algorithms.

**Table 1:** Differentially expressed genes (DEGs) between malignant and non-malignant cells commonly in five cancer single-cell transcriptome datasets.

DEGs	Gene symbol
upregulated genes in malignant cells (TMGs)	*SERINC2, PTPRF, S100A16, EFNA1, SOX4, PERP, HSPB1, ASPH, LAPTM4B, BNIP3, EPS8L2, CCND1, CTTN, KRT18, CKAP4, CKB, SEMA4B, TNFRSF12A, CRNDE, GCSH, DMKN, SDC4, CDC42EP1, HOOK2, TMEM54, UGDH, TFG, FLNB, ENO1, P4HB, S100A10, TRIP6, SCD, TMEM9, SPR, TM4SF1, MIF, ARPC1A, HIST1H1C, HDGF, POLD2, AHCY, PAFAH1B3, POP7, NHP2, PDIA4, CKS1B, ALDOA, LTBR, TMEM141, PSMB5, WDR34, HSBP1L1, AP1S1, PDCD5, NDUFB4, H1F0, HSBP1, TPI1, CTNND1, SEC61G, SPINT1, AGPAT2, LGALS3BP, POLR2I, UQCRQ, TIMM13, NDUFC1, COX6C, SNRPE, NDUFB9, PRDX5, ANXA2*
upregulated genes in non-malignant cells (NMGs)	*TNFRSF1B, CD52, LAPTM5, CD53, CD2, CTSS, S100A4, RCSD1, RGS1, PTPRC, ZFP36L2, CXCR4, ARHGAP15, CYTIP, WIPF1, HCLS1, GZMA, HLA-A, HLA-E, HLA-C, HLA-B, GPSM3, HLA-DQA1, HLA-DPB1, IKZF1, GIMAP4, SLA, SRGN, LSP1, CTSW, UCP2, CD69, ARHGDIB, BIN2, GLIPR1, ALOX5AP, LCP1, EVL, B2M, CORO1A, CYBA, ACAP1, EVI2B, EVI2A, CCL5, CCL4, FMNL1, DDX5, CD7, MYO1F, FXYD5, HCST, GMFG, CD37, NKG7, CST7, STK4, SAMSN1, ITGB2, PIK3IP1, TMSB4X, IL2RG, CALM1, ABI3, GIMAP7, CD3D, TRAF3IP3, RHOG, CD247, IL10RA, LCK, PSMB9, FNBP1, CD3E, STK17B, FGL2, PPP1R18, RGS2, CD4, ARL6IP5, GPR65, FERMT3, LCP2, GPR183, BTG1, RHOH, ANKRD44, DUSP2, PTPN7, SEPT6, LYZ, TBC1D10C, DOK2, PARVG, AKNA, GLIPR2, FYN, SMAP2, CREM, CD96, PRDM1, ENTPD1, GZMK, ITM2A, CD3G, HIF1A, EEF1A1, LTB, DOCK8, CLEC2D, PSME1, FOXP1, SELPLG, RUNX3, RILPL2, PTPN22, PTPN6, SYTL3, STAT4, PTGER4, IL16, RASSF5, CD27, FLI1, CD8A, TAGAP, APBB1IP, NCF4, PPP2R5C, STX11, DEF6, ARHGAP9, RASAL3, SASH3, INPP5D, EPSTI1, LIPA, RGCC, PSMB10, RGS19, CTSC, ARID5A, ETS1, LPXN, SPOCK2, TCF4, STK17A, CCND2, SERPINB9, AKAP13, SH2D1A, CD74, ARHGEF1, IL7R, TNFAIP3, MFNG, ZNF331, SH2D2A, CARD8, ISG20, CSK, CNOT6L, USP15, ARHGAP4, PIM1, PPM1K, DAZAP2, SRSF5, ZFP36, AAK1, TERF2IP, JUNB, PLK3, FGFR1OP2, NFKBIA, ICAM3, ARL4C, SKAP1, IRF1, ANXA6, G3BP2, TNFAIP8, GALM, CFD, SEPT1, CCDC85B*

### 3.1 The DEGs have significant associations with cancer-related molecular and clinical features in pan-cancer

**Fig. 2:**
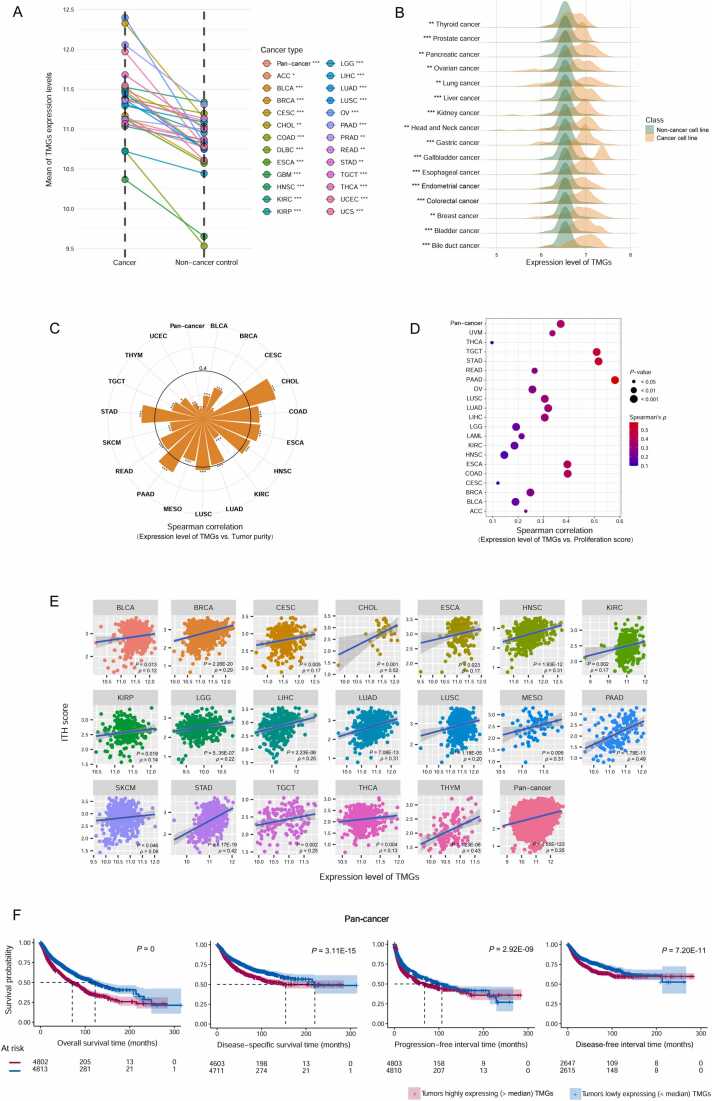
**Correlations between the expression of tumor marker genes (TMGs) and molecular and clinical features in pan-cancer. A** TMGs showing significantly higher expression levels in TCGA pan-cancer and in 25 individual cancer types than in normal controls. **B** TMGs showing significantly higher expression levels in 16 types of cancer cell lines than in normal controls. **C–E** TMGs having significant positive expression correlations with tumor purity, proliferation scores, and intratumor heterogeneity (ITH) scores in pan-cancer and in multiple individual cancer types. **F** The tumors with higher expression (> median) of TMGs displaying significantly worse prognosis than the tumors with lower expression (< median) of TMGs in pan-cancer. Student’s *t* test FDR are shown in **A** and **B**, with * FDR < 0.05, ** FDR < 0.01*,* *** FDR < 0.001. Spearman correlation coefficients *(ρ*) and *P*-values are shown in **C–E**, with *P < 0*.*05, **P < 0.01, ***P < 0.001. Log-rank test *P*-values are shown in **F**.

The DEGs included 73 TMGs and 186 NMGs. We defined the expression level of a gene set in a sample as the average expression level of all genes in the gene set. Notably, compared to normal controls, TMGs displayed significantly higher expression levels in TCGA pan-cancer (*P* = 2.22 × 10^−39^) and in 25 of the 30 individual cancer types with normal sample’s size not less than five (FDR < 0.05) (Fig. 2A). Moreover, TMGs were significantly upregulated in 16 of 21 types of cancer cell lines analyzed versus normal controls (FDR < 0.05) (Fig. 2B). In addition, TMGs showed significant positive expression correlations with tumor purity in pan-cancer and in 18 individual cancer types (*P* < 0.05) (Fig. 2C). The expression of TMGs correlated positively with proliferation scores in pan-cancer and in 20 individual cancer types (*P* < 0.05) (Fig. 2D). Furthermore, the expression of TMGs correlated positively with ITH scores in pan-cancer and in 19 individual cancer types (*P* < 0.05) (Fig. 2E). These results collectively support that the TMGs we identified are authentic markers of cancer cells. Furthermore, we observed that the tumors with high expression (> median) of TMGs had significantly worse prognosis than the tumors with low expression (< median) of TMGs in pan-cancer (log-rank test, *P* = 0, 3.1 × 10^−15^, 2.9 × 10^−9^ and 7.2 × 10^−11^ for overall survival (OS), disease-specific survival (DSS), progression-free interval (PFI) and disease-free interval (DFI), respectively) (Fig. 2F). In addition, in 11 individual cancer types, the tumors highly expressing (> median) TMGs had significantly lower OS and/or disease-free survival (DFS) rates than the tumors lowly expressing (< median) TMGs (*P* < 0.05) (Supplementary Fig. S1). Again, these results support the role of cancer markers of the TMGs.

**Fig. 3:**
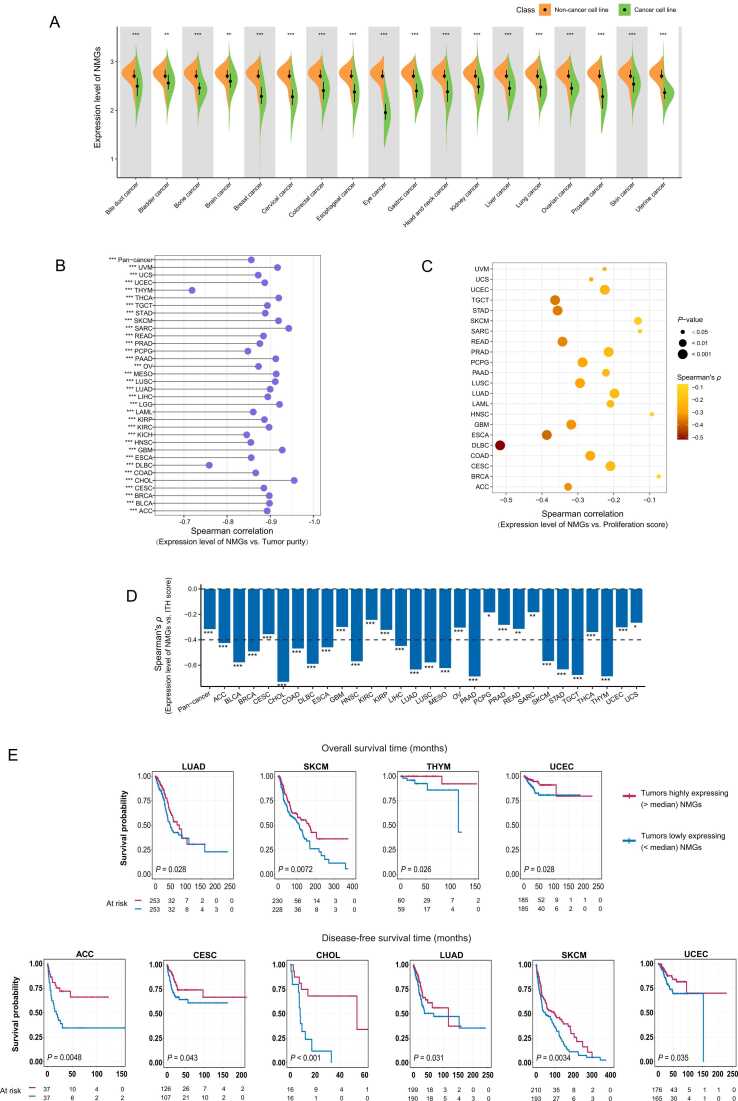
**Correlations between the expression of non-tumor marker genes (NMGs) and molecular and clinical features in pan-cancer. A** NMGs showing significantly lower expression levels in 18 types of cancer cell lines than in normal controls. **B–D** NMGs having significant negative expression correlations with tumor purity, proliferation scores, and intratumor heterogeneity (ITH) scores in pan-cancer and in multiple individual cancer types. **E** The tumors with higher expression (> median) of NMGs displaying significantly better overall and/or disease-free survival prognosis than the tumors with lower expression (< median) of NMGs in seven cancer types. Student’s *t* test FDR are shown in A, with * FDR < 0.05, ** FDR < 0.01*,* *** FDR < 0.001. Spearman correlation coefficients *(ρ*) and *P*-values are shown in **B–D**, with *P < 0*.*05, **P < 0.01, ***P < 0.001. Log-rank test *P*-values are shown in **E**.

On the contrary to TMGs, NMGs showed significantly lower expression levels in 18 types of cancer cell lines compared to normal controls (FDR < 0.05) (Fig. 3A). The expression of NMGs had strong negative correlations with tumor purity in pan-cancer and in all 33 individual cancer types (*ρ* < − 0.70) (Fig. 3B), while it showed strong positive correlations with immune scores in pan-cancer and in all 33 individual cancer types (*ρ* ≥ 0.89) (Supplementary Fig. S2A). Moreover, NMGs displayed significant positive correlations with stromal scores in pan-cancer and in most individual cancer types (*P* < 0.001; *ρ* > 0.27) (Supplementary Fig. S2B). These results are justified since NMGs are significantly upregulated in non-malignant cells, which involves immune and stromal cells. As opposed to TMGs, NMGs displayed significant negative expression correlations with proliferation scores in 22 individual cancer types (*P* < 0.05) (Fig. 3C). The expression of NMGs correlated negatively with ITH scores in pan-cancer and in 29 individual cancer types (*P* < 0.05) (Fig. 3D). Taken together, these results support that the NMGs are markers of non-cancer cells. Notably, the tumors with high expression (> median) of NMGs had significantly higher OS and/or DFS rates than the tumors with low expression (< median) of them in seven individual cancer types (*P* < 0.05) (Fig. 3E).

**Fig. 4:**
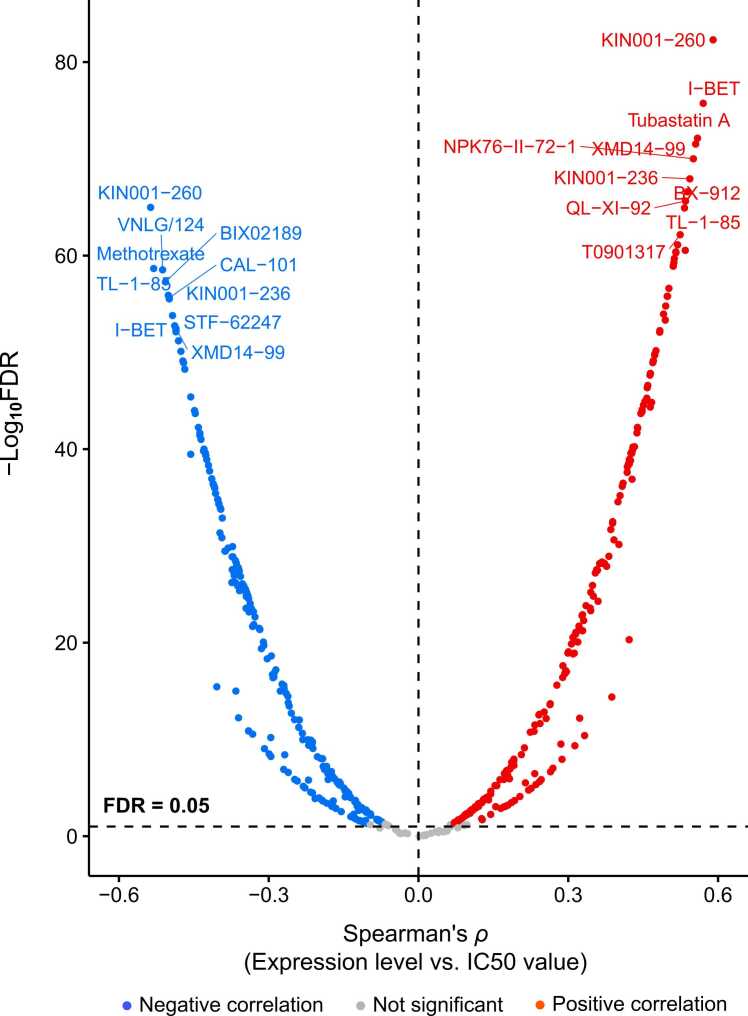
**Spearman correlations between the expression of TMGs and NMGs and IC50 values of 265 compounds in cancer cell lines.** The significant positive and negative correlations (FDR < 0.05) are indicated in red and blue, respectively. Among 265 compounds tested in cancer cell lines, the expression of TMGs shows significant positive correlations with IC50 values in 212 compounds highlighted in red, and the expression of NMGs has significant negative correlations with IC50 values in 232 compounds highlighted in blue.

We further analyzed correlations between the expression of DEGs and drug sensitivity (IC50 values) in cancer cell lines using the data from the Genomics of Drug Sensitivity in Cancer (GDSC) project (https://www.cancerrxgene.org). Interestingly, among 265 compounds tested in cancer cell lines, the expression of TMGs showed significant positive correlations with IC50 values in 212 (80 %) compounds, and the expression of NMGs had significant negative correlations with IC50 values in 232 (88 %) compounds (FDR < 0.05) (Fig. 4 and Supplementary Table S2). These results indicate that upregulation of TMGs is associated with reduced drug sensitivity and that upregulation of NMGs is associated with increased drug sensitivity in cancer.

### 3.2 PreCanCell can accurately classify malignant and non-malignant cells in various cancer types

**Fig. 5:**
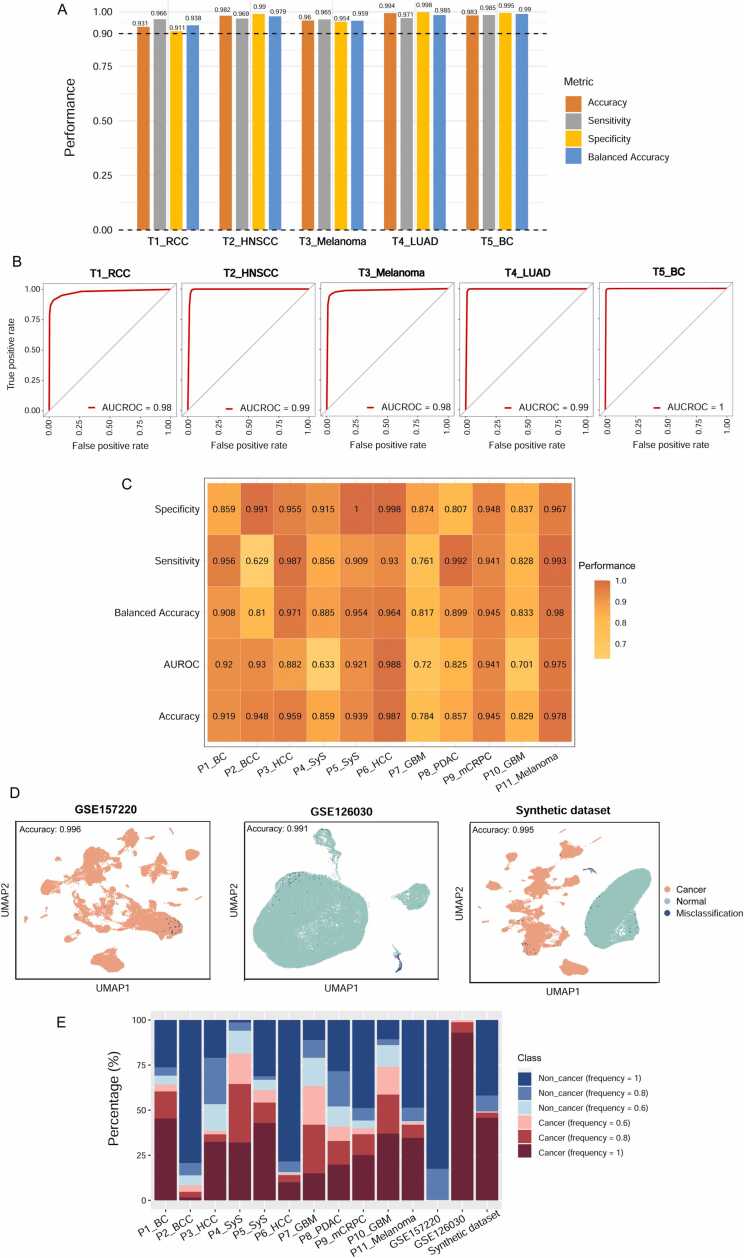
**Predictive performance of the PreCanCell algorithm. A, B** The predictive performance of the *k*-NN (*k* = 5) classifier with the TMGs and NMGs as features in the five training datasets. In each of the five training datasets, the 10-fold cross-validation accuracy, sensitivity, specificality, balanced accuracy, and AUROC are shown. **C** Heatmap showing the predictive performance of PreCanCell in 11 test sets. **D** The predictive performance of PreCanCell in three datasets, which were composed of solely malignant cells (GSE157220), solely normal cells (GSE126330), and both malignant and normal cells, respectively. **E** The voting ratios of cancer or non-cancer cells by the five base *k*-NN classifiers in each dataset. Frequency represents the voting ratio of the five base classifiers predicting a cell as malignant or non-malignant.

We first tested the predictive performance of the *k*-NN classifier with the DEGs as features in the five training datasets. In each of the five training datasets, the 10-fold cross-validation accuracy, sensitivity, specificality, balanced accuracy (Fig. 5A), and AUROC (Fig. 5B) were all greater than 0.90. The high predictive performance in the training datasets was expected since the predicted datasets were involved in feature selection. We further tested the PreCanCell algorithm in 11 independent test sets. These test sets were single-cell transcriptome datasets for BC, BCC, HCC, SyS, GBM, PDAC, mCRPC and melanoma, respectively. Among the 11 test sets, the prediction accuracy was more than 0.90 in seven datasets and between 0.80 and 0.90 in three datasets (Fig. 5C); the balanced accuracy was greater than 0.80 in all the 11 test sets and more than 0.90 in six test sets. The sensitivity was greater than 0.80 in nine test sets and more than 0.90 in seven test sets; the specificity was greater than 0.80 in all the 11 test sets and more than 0.90 in seven test sets. Finally, the AUROC was greater than 0.80 in eight test sets and more than 0.90 in six test sets. Of note, PreCanCell showed superior predictive performance not only in the test sets whose cancer type was involved in the training set, but also in the test sets whose cancer type was not involved in the training set, such as BCC, HCC, SyS, GBM, PDAC and mCRPC (Fig. 5C). Taken together, these results suggest excellent performance of PreCanCell in classifying malignant and non-malignant cells in various cancer types.

We further tested PreCanCell using three additional datasets, which were composed of solely malignant cells, solely normal cells, and both malignant and normal cells, respectively. In the dataset GSE157220 only containing malignant cells, 222 out of 53,513 cancer cells were predicted as normal cells, with a false negative rate of 0.0041 (Fig. 5D). In the dataset GSE126030 only containing normal cells, 566 out of 63,861 normal cells were predicted as cancer cells, with a false positive rate of 0.0089. The third synthetic dataset was created by artificially combining GSE157220 and GSE126030. In this dataset, PreCanCell also achieved high predictive performance, with 0.995 accuracy, 0.998 sensitivity, 0.993 specificality, 0.995 balanced accuracy and 0.995 AUROC.

PreCanCell is an ensemble algorithm that uses the majority voting principle. To observe how agreeable the five base classifiers, we recorded the classification results of each base classifier in each dataset (Fig. 5E). In P6_HCC, P11_Melanoma, GSE157220, GSE126030 and the synthetic dataset, the five base classifiers displayed a high agreement in predicting malignant and non-malignant cells. Accordingly, PreCanCell achieved better predictive performance in these datasets. In contrast, in P4_SyS, P7_GBM and P10_GBM, the five base classifiers achieved relatively inconsistent results, and thus PreCanCell showed relatively poorer performance.

### 3.3 PreCanCell achieves better predictive performance than most other algorithms

**Fig. 6:**
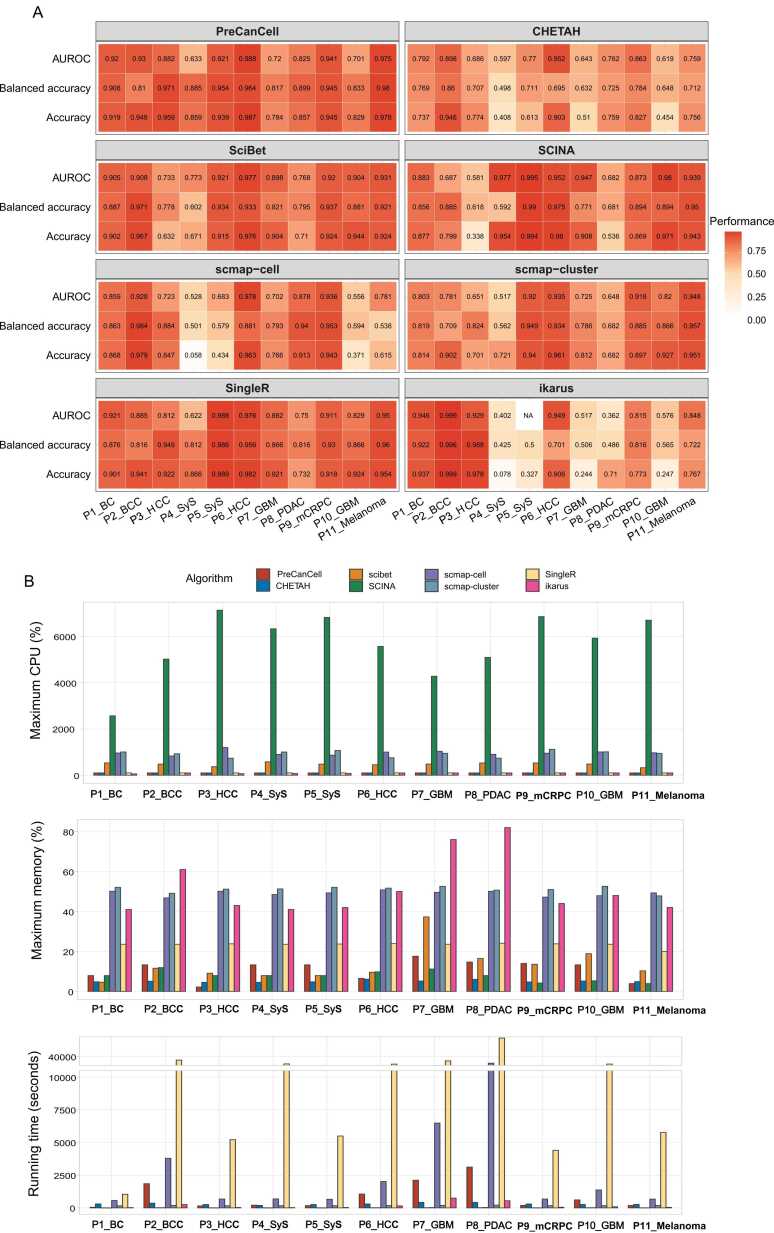
**Comparisons of the predictive performance between PreCanCell and seven other algorithms. A** The AUROC, accuracy, and balanced accuracy of the eight algorithms in predicting malignant and non-malignant cells in 11 test sets. **B** The maximum CPU usage, maximum memory usage and running time required by the eight algorithms in predicting malignant and non-malignant cells in 11 test sets.

We compared the predictive performance (AUROC, accuracy and balanced accuracy) of PreCanCell with seven other algorithms in predicting malignant and non-malignant cells in the 11 test sets (Fig. 6A). These algorithms included CHETAH [7], SciBet [8], SCINA [9], scmap-cell [10], scmap-cluster [10], SingleR [11] and ikarus [12]. Here we chose three metrics (AUROC, balanced accuracy and accuracy) instead of the previous five, because the balance accuracy is the arithmetic mean of sensitivity and specificity, which together with AUROC and accuracy are sufficient in comparing algorithm’s performance. In addition, it is more concise to display the numerous results generated by eight algorithms in 11 test sets with the three metrics. Notably, PreCanCell achieved the greatest AUROC in three datasets and the second greatest AUROC in four datasets; PreCanCell had the highest accuracy in three datasets and the second highest accuracy in three datasets; PreCanCell had the highest balanced accuracy in two datasets and the second highest balanced accuracy in six datasets.

We also compared the computational resource and running time required by these algorithms (Fig. 6B). Of note, PreCanCell required less computational resource than most of the seven existing algorithms. For example, when running the algorithms in the 11 test sets, PreCanCell had the maximum CPU usage of 101 %, the same as CHETAH, SingleR and ikarus but much less than the other algorithms; the maximum memory required by PreCanCell overall exceeded CHETAH and SCINA but less than the other algorithms. As for running time, PreCanCell was faster than scmap-cell and SingleR, close to CHETAH and scmap-cluster and slower than SciBet, SCINA and ikarus. It is worth noting that PreCanCell is faster than CHETAH in small datasets (such as P1_BC, P3_HCC, P5_SyS, P9_mCRPC and P11_Melanoma), but slower than CHETAH in large datasets (such as P2_BCC, P6_HCC, P7_GBM, P8_PDAC and P10_GBM). It is reasonable as *k*-NN is a lazy learning algorithm that focuses on prediction rather than training.

## 4 Discussion

This study proposes a novel algorithm: PreCanCell, to predict cancer and non-cancer cells from single-cell transcriptomes. Compared to the established algorithms for cell type annotation, PreCanCell has several advantages. First, it is more accurate than most algorithms in predicting malignant and non-malignant cells. Its excellent predictive performance is mainly attributed to: (1) the use of well-annotated and high-quality cancer single-cell transcriptomes as the training set; and (2) the use of an ensemble learning algorithm for class prediction. PreCanCell has overcome the drawback of many methods developed earlier using poorly-annotated training sets or reference sets. In addition, PreCanCell employs ensemble learning that has been shown to be more accurate and robust than a single model [30]. Indeed, we have tried to predict malignant and non-malignant cells in the test set P1_BC with a single training dataset T5_BC by *k*-NN, both of which were breast cancer single-cell transcriptomes. We obtained 0.835, 0.777, 0.930, 0.853 and 0.834 of accuracy, sensitivity, specificality, balanced accuracy and AUROC, respectively, compared to 0.919, 0.956, 0.859, 0.908 and 0.92 of them by PreCanCell. It proves that PreCanCell has significantly better performance than the single model. The second advantage of PreCanCell is its simplicity. PreCanCell does not require users to provide gene markers and training or reference datasets. In addition, it is implemented with a single R package. Furthermore, as *k*-NN is a lazy learning algorithm that focuses on prediction rather than learning or training, the implementation of PreCanCell is simple and straightforward. Users can even add additional training datasets to improve PreCanCell’s prediction performance. The third advantage of PreCanCell is that it needs less computational resources, such as CPU and memory usage, than most existing algorithms. Finally, PreCanCell is set up with parallel operations so that it can be implemented with multiple workers to increase running speed. It is particularly useful in predicting large datasets for which *k*-NN is relatively time-consuming.

Here we chose *k*-NN as the base classifier for its some advantages over other algorithms. First, *k*-NN is a non-parametric method that does not make any assumption about data distribution; thus, *k*-NN is suitable for various types of data, including complex and non-linear data. Unlike bulk transcriptomes, single-cell transcriptomes are not normally distributed for which *k*-NN is a viable method. Second, *k*-NN is robust to noise and outliers since it uses a majority vote to reduce the impact of mislabeled samples. Again, it is quite suitable for single-cell transcriptomes which are full of noise. Finally, as a lazy learning algorithm, taking high-quality training sets as background, *k*-NN often can achieve a high prediction performance. Here we set *k* = 5 because: (1) for binary classification, the *k* of *k*-NN should be an odd number; (2) the *k*-NN’s prediction performance was the best in the training datasets when *k* ranged from 1 to 15; and (3) when *k* = 5, the algorithm’s computational complexity is lower than that with *k* > 5.

In some datasets, some of the compared algorithms have achieved inferior performance (Fig. 6A). A main reason behind this could be that most of these algorithms, including CHETAH, SciBet, scmap-cell, scmap-cluster, SingleR and SCINA, were not developed specifically for annotating cancer cells. Only ikarus is a tool specifically designed to identify malignant cells. Nevertheless, we observed that ikarus had poor predictive performance in several datasets, such as P4_SyS, P5_SyS, P7_GBM, P8_PDAC and P10_GBM (Fig. 6A). We contend that the feature genes selected by these algorithms are not highly adequate to discriminate between malignant and non-malignant cells. Specifically, our algorithm selects the feature genes which are consistently upregulated in malignant or non-malignant cells across five common cancer types. As a result, our feature genes display a stronger power to separate malignant from non-malignant cells.

In addition to develop the novel algorithm for cancer cell identification, this study uncovered cancer marker genes by analyzing single-cell transcriptome datasets for five common cancer types: RCC, HNSCC, BC, LUAD and melanoma. We further demonstrated that the cancer marker genes had significant associations with malignancy-related characteristics in tumor bulk and cancer cell lines, such as their upregulation correlated with heightened tumor proliferation capacity, ITH and drug resistance, and poor prognosis in pan-cancer and in diverse individual cancer types.

There are several limitations in this study. First, the reported predictive performance in the training set could be overestimated due to the predicted sets being involved in feature selection. Nevertheless, the reported predictive performance in the test sets should be warranted. Second, this algorithm is limited to annotating cancer and non-cancer cells, while it is not designed for identifying subpopulations of non-cancer cells, such as immune cells, stromal cells and epithelial cells. This is a direction for us to extend the algorithm in the future.

## Ethical approval

Ethical approval and consent to participate were waived since we used only publicly available data and materials in this study.

## CRediT authorship contribution statement

**Tao Yang:** Data curation, Formal analysis, Investigation, Software, Validation, Writing - review & editing, Visualization. **Qiyu Yan:** Visualization, Data curation, Formal analysis, Validation. **Rongzhuo Long:** Visualization, Formal analysis, Software. **Zhixian Liu:** Project administration, Funding acquisition, Investigation. **Xiaosheng Wang:** Conceptualization, Methodology, Resources, Investigation, Writing - original draft, Supervision, Project administration.

## Funding

This work was supported by 10.13039/501100004608Natural Science Foundation of Jiangsu Province (BK20201090) and 10.13039/501100002858China Postdoctoral Science Foundation (2021M691338) to ZL.

## Declaration of Competing Interest

The authors declare that they have no competing interests.

## Data Availability

All data supporting the findings of this study are available within the paper and its Supplementary information. The R package for the PreCanCell algorithm is available at https://github.com/WangX-Lab/PreCanCell.

## Appendix A Supplementary material

**Figure S1.** The tumors highly expressing (> median) TMGs had significantly lower overall and/or disease-free survival rates than the tumors lowly expressing (< median) TMGs in 11 individual cancer types.

**Figure S2. Correlations between the expression of non-tumor marker genes (NMGs) and immune and stromal signatures.** NMGs having significant positive expression correlations with immune scores (**A**) and stromal scores (**B**) in pan-cancer and in most individual cancer types. Spearman correlation coefficients (*ρ*) and *P*-values are shown in **A** and **B**.
