# Supplementary material for: PreCanCell: An ensemble learning algorithm for predicting cancer and non-cancer cells from single-cell transcriptomes
Source: Comput Struct Biotechnol J. 2023 Jul 11;21:3604–14. doi: 10.1016/j.csbj.2023.07.009 (PMC10371765; doi:10.1016/j.csbj.2023.07.009)
Supplement: Supplementary file 1 — Figure S1. The tumors highly expressing (> median) TMGs had significantly lower overall and/or disease-free survival rates than the tumors lowly expressing (< median) TMGs in 11 individual cancer types. [file mmc1.pdf]

Fig S1

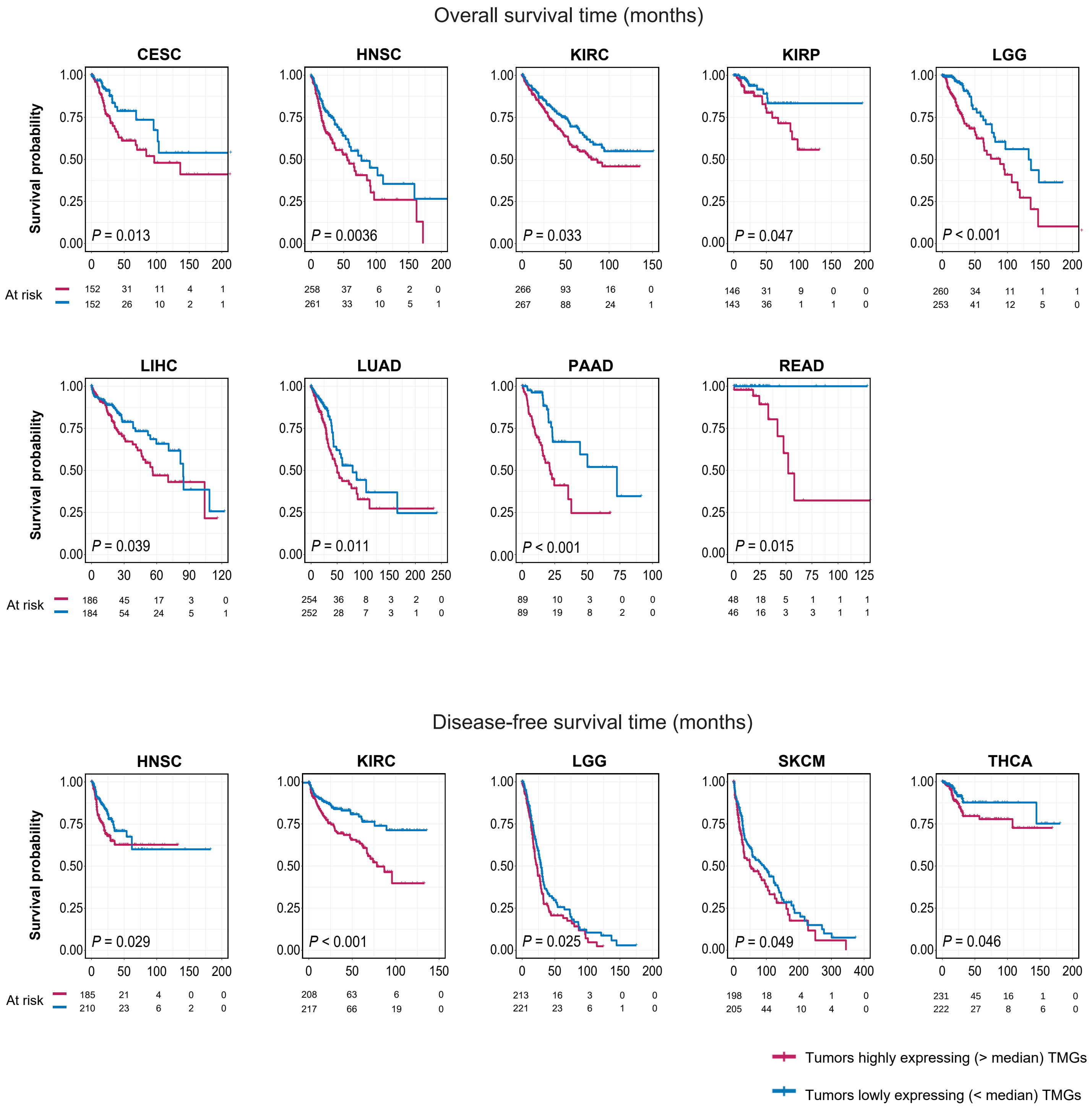

Overall survival time (months)
CESC
HNSC
KIRC
KIRP
LGG
Survival probability
P = 0.013
P = 0.0036
P = 0.033
P = 0.047
P < 0.001
At risk
152 31 11 4 1
152 26 10 2 1
258 37 6 2 0
261 33 10 5 1
266 93 16 0
267 88 24 1
146 31 9 0 0
143 36 1 1 0
260 34 11 1 1
253 41 12 5 0
LIHC
LUAD
PAAD
READ
P = 0.039
P = 0.011
P < 0.001
P = 0.015
186 45 17 3 0
184 54 24 5 1
254 36 8 3 2 0
252 28 7 3 1 0
89 10 3 0 0
89 19 8 2 0
48 18 5 1 1 1
46 16 3 3 1 1
Disease-free survival time (months)
HNSC
KIRC
LGG
SKCM
THCA
P = 0.029
P < 0.001
P = 0.025
P = 0.049
P = 0.046
185 21 4 0 0
210 23 6 2 0
208 63 6 0
217 66 19 0
213 16 3 0 0
221 23 6 1 0
198 18 4 1 0
205 44 10 4 0
231 45 16 1 0
222 27 8 6 0
Tumors highly expressing (> median) TMGs
Tumors lowly expressing (< median) TMGs
