# Supplementary figures and images for: PreCanCell: An ensemble learning algorithm for predicting cancer and non-cancer cells from single-cell transcriptomes
Source: Comput Struct Biotechnol J. 2023 Jul 11;21:3604–14. doi: 10.1016/j.csbj.2023.07.009 (PMC10371765; doi:10.1016/j.csbj.2023.07.009)

Fig S2

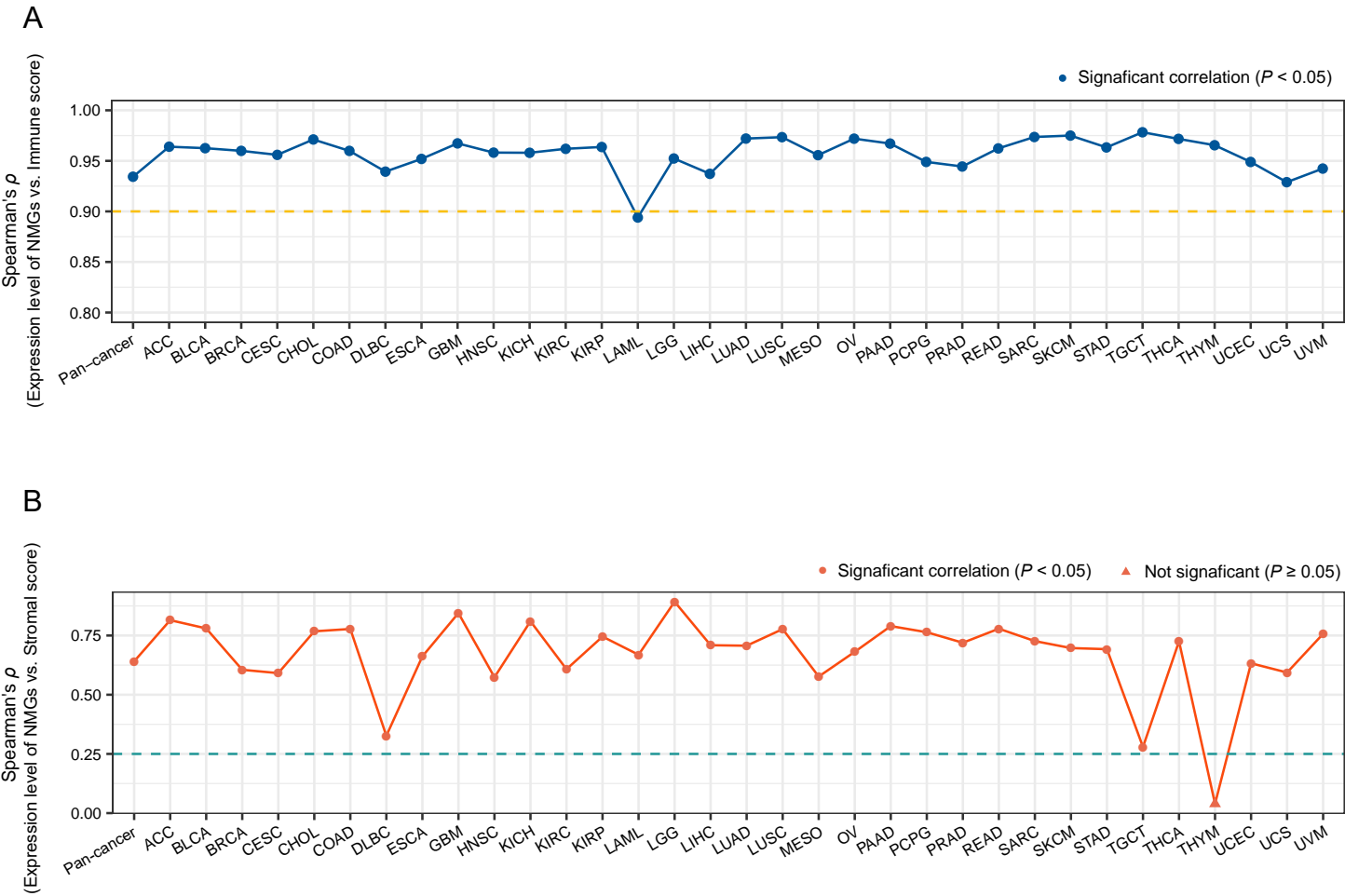

Supplement: Supplementary file 2 — Figure S2. Correlations between the expression of non-tumor marker genes (NMGs) and immune and stromal signatures. NMGs having significant positive expression correlations with immune scores (A) and stromal scores (B) in pan-cancer and in most individual cancer types. Spearman correlation coefficients (ρ) and P-values are shown in A and B. [file mmc2.pdf]
